# Comorbidities and Disease Duration in Tourette Syndrome: Impact on Cognition and Quality of Life of Children

**DOI:** 10.3390/children11020226

**Published:** 2024-02-09

**Authors:** Giulia Conte, Carola Costanza, Maria Novelli, Veronica Scarselli, Elena Arigliani, Francesca Valente, Valentina Baglioni, Arianna Terrinoni, Flavia Chiarotti, Francesco Cardona

**Affiliations:** 1 Child and Adolescent Neuropsychiatry Unit, Department of Human Neuroscience, Sapienza University of Rome, 00185 Rome, Italy; giulia.conte@uniroma1.it (G.C.); maria.novelli@uniroma1.it (M.N.); veronicascarselli@gmail.com (V.S.); ele.arigliani@gmail.com (E.A.); francescavalente87@gmail.com (F.V.); valentina.baglioni@uniroma1.it (V.B.); a.terrinoni@policlinicoumberto1.it (A.T.); 2 Department of Sciences for Health Promotion and Mother and Child Care “G. D’Alessandro”, University of Palermo, 90128 Palermo, Italy; carola.costanza@unipa.it; 3 Center for Behavioral Sciences and Mental Health, Istituto Superiore di Sanità, 00161 Rome, Italy; flavia.chiarotti@iss.it

**Keywords:** Tourette syndrome, comorbidities, disease duration, cognition, cognitive profile, quality of life, obsessive compulsive disorder (OCD), attention-deficit/hyperactivity disorder (ADHD), depression, emotional dysregulation

## Abstract

Background: Cognitive functions represent foundational factors for mental health and quality of life (QoL). In Tourette syndrome (TS), psychiatric comorbidities are common and have been inconsistently reported to affect the cognition and QoL of patients, while the role of tic disorder duration has not been yet explored. Methods: To examine how comorbidities and TS duration may influence cognition and QoL, *N* = 80 children with TS (6–16 years) were evaluated using the Wechsler Intelligence Scale for Children (WISC-IV). Standardized questionnaires were used to assess the presence and severity of TS main comorbidities and QoL. Data were interpreted using linear correlations, regression, and mediation analysis. Results: Depression and attention-deficit/hyperactivity disorder (ADHD) symptoms accounted for poorer cognitive performance. Anxiety oppositely predicted better cognitive performance, while no significant role for obsessive compulsive disorder (OCD) was observed. Disease duration was associated with lower total IQ, verbal reasoning, and working memory abilities. Depression, anxiety, and TS duration also deeply influenced QoL measures. Conclusions: TS common comorbidities have a differential impact on the cognitive abilities of children and adolescents, which translates into a complex influence on their perceived QoL. A longer clinical history of tics was related to worse cognitive outcomes, which prompts further consideration of disease duration in both clinical and research settings involving children and adolescents.

## 1. Introduction

Tourette syndrome (TS) is a complex childhood-onset neuropsychiatric condition characterized by motor and vocal tics—i.e., repetitive, sudden, involuntary movements or vocalizations—which occur for at least one year [1]. Although the majority of people living with mild forms of TS lead satisfying lives, those with greater tic severity and persistent symptoms may experience a significant negative impact on their overall quality of life and well-being [2].

There is extreme variability in the clinical presentation of TS [3,4] and in the associated clinical conditions, i.e., comorbidities, that are usually developed by two-thirds of affected individuals and often arise during childhood or early adolescence [5,6,7]. Comorbidities include several neurodevelopmental and psychiatric disorders such as attention-deficit/hyperactivity disorder (ADHD) [6], obsessive compulsive disorder (OCD) and other repetitive behaviors [8,9], depression [10,11], and anger dysregulation with disruptive behaviors [12].

Coupled with disabling and long-lasting tics, comorbidities can all lead to poorer psychosocial functioning and quality of life (QoL) [13,14]. Comorbid disorders have been linked to greater TS severity [3] and a greater load of socially inappropriate behaviors which fall within the spectrum of complex tics (such as copropraxia or coprolalia), potentially leading to poor psychosocial adjustment [15]. Comorbid disorders are also associated with greater TS duration [4] and a greater impact on health-related QoL [14,16]. However, the effect of comorbidities on QoL extends beyond their association to more severe tic phenotypes. Depression, anxiety, OCD, and ADHD not only act on well-being through the psychopathological mechanisms inherent to each one of them (e.g., low self-esteem in anxiety and depression, poor frustration tolerance and impulsivity in ADHD and disruptive behaviors, leading to social isolation and exclusion or poor academic progress), but are also significantly burdened by variable but enduring impairments in cognition, and specifically in attention, memory, and executive functions [17,18,19,20,21,22].

Cognitive functions, by enabling individuals to categorize and remember information; maintain attention for reasoning; adapt responses to changes in the environment, personal goals, etc.; represent foundational factors to learn and attain education [23,24]; reach a successful transition to adulthood [25]; and, most importantly, maintain good mental health [26,27]. While intelligence is generally considered to fall within the average range in TS [28], several studies point to minor neuropsychological deficits regarding inhibition, cognitive flexibility, and working memory [29,30,31], while others conversely support the presence of enhanced cognitive abilities with respect to procedural learning [32] and inhibitory control [33,34]. Thus, no conclusive evidence is available to date regarding cognition in TS and how this is affected by the different combination of TS’s typical comorbidities. Nonetheless, investigating cognition in neuropsychiatric disorders appears relevant for patients in all levels of care. Interestingly, in depressive and psychotic spectrum disorders, cognitive functioning is a predictor of the illness course in the long-term, independently from the variation of symptoms defining the clinical picture (e.g., hallucinations in schizophrenia, mood regulatory problems in depression) [35,36]. Moreover, cognition is not only increasingly recognized as a key determinant for well-being throughout developmental ages and later in life [37,38] but may also represent a promising target for novel treatments aimed at training cognitive functioning with the ultimate goal of improving functional outcomes and QoL—the so-called cognitive remediation strategies [39,40,41,42]. Regarding comorbidities, while ADHD has been extensively reported to negatively influence cognition in the specific context of TS [43,44,45,46,47,48], data on how OCD, anxiety, and depression may impact the cognitive functioning of people with chronic tics are much scarcer, at best. Additionally, evidence on the extent to which each different comorbidity moderates cognitive performance in TS remains hampered by the categorial approaches implemented by prior studies, which parse the clinical spectrum into classes or groups (e.g., TS + ADHD vs. TS + OCD vs. TS + ADHD + OCD, etc.). These have the downside of creating simplistic differentiations of the clinical picture of TS, possibly lacking ecologic validity for a nuanced spectrum in which comorbidities combine differently from one patient to another, both in terms of type and severity.

Not only do comorbidities have enormous interindividual variation in TS, but tics also do. Tics may also dramatically change during the life course of TS for the single affected person, both in severity and persistence over time. Prior studies showed that a younger age at tic onset [44,49] and greater tic severity [49,50] seem to negatively influence cognitive abilities, although such effects were not confirmed in larger population-based groups of children [51]. As for the persistence of tics over time, little is currently known about how this variable may influence the QoL of people with TS, and in which way it may do so. Although disease duration has been scantly considered in the clinical literature around TS, having to deal with more persistent tics or being under the burden of repeating episodes of tic exacerbations is highly likely to play a role in the well-being and functioning of people living with TS. Still, it is unknown whether a greater disease duration may harm patients’ QoL by acting on their cognitive functioning as much as other tic-related factors previously outlined.

Against this background, studies aimed at evaluating if and to what extent the variable clinical severity of TS influences cognition and QoL appear of critical importance to promote optimal care for all patients, and children particularly. Prior TS studies have approached this issue, providing mixed results, which may have arisen from TS’s typical heterogeneity. Therefore, the present study aimed to explore how much tic-related clinical aspects, on one side, and comorbid disease load, on the other, impact the cognitive functioning of children with TS and if this is relevant to their emotional, academic, and social functioning and overall well-being. More specifically, we implemented a dimensional approach that accounts for the variability in tic and comorbid disease severity on cognitive performance and QoL, without parsing the clinical spectrum of TS into rigid categories (e.g., TS + depression vs. TS + ADHD vs. TS + OCD, and so on) that may fall short of ecological validity [4] and add little to clinical practice. To our knowledge, this is the first study to address cognitive functioning and QoL in TS by means of a dimensional approach in order to reveal the independent contribution of each major TS comorbidity and tic persistence over time (i.e., disease duration). Based on previous evidence, we assume that lower cognitive performances may be related to poorer school functioning and quality of life in children with TS. We also hypothesize that ADHD symptom severity may be strongly associated with multiple deficits in multiple cognitive domains, while we do not pose any peculiar hypothesis as regards the role of comorbid OCD, anxiety, and depression, given the heterogeneous or lacking data from the prior literature. Further, along with the increasing recognition that disease duration is associated with worse clinical outcomes in conditions proximal to TS, such as OCD [52], we speculate that a longer tic duration may negatively impact cognition and point out that the issue of disease duration may require further consideration by studies with clinical populations. Lastly, we aimed to unravel if cognitive functioning mediates the effect of comorbidities on QoL in TS to inform both diagnostic and treatment strategies for pediatric patients.

## 2. Materials and Methods

The outpatient clinic for TS and associated disorders at the Sapienza University of Rome provides evaluation and treatment for children and adolescents with TS and disorders of the impulsive–compulsive spectrum. All children undergo an intake visit consisting of a comprehensive anamnesis, a neurological examination, and a few clinical assessments, including the Yale Global Tic Severity Scale (YGTSS) [53] and the Child Behavior Checklist [54] to screen behavioral and emotional problems. Fully qualified child and adolescent neuropsychiatrists with adequate experience in TS then formulate diagnoses according to the DSM-5 [1]. Thereafter, all patients diagnosed with TS are offered a comprehensive assessment which includes neuropsychological testing and an evaluation of comorbid conditions to deliver appropriate treatment for each patient- and family-specific need.

### 2.1. Participants

Eighty children with TS (72 boys and 8 girls) aged 6–16 years were consecutively recruited between January 2017 and March 2021 at our specialty clinic. All study data were drawn from a cumulative clinical sample of children seen for the first time at our clinic for the standard diagnostic protocol in the timeframe considered for the study. Thus, we did not operate a sample selection based on clinical characteristics to allow for a greater representativeness of patients routinely followed at our site. The participants’ data were all anonymized and stored in an offline electronic repository accessible only to the study researchers. Due to anonymous data collection, informed consent from participants was not required. This study was approved by the institutional review board of the Sapienza University of Rome and performed in accordance with the Declaration of Helsinki.

#### 2.1.1. Inclusion Criteria

The following criteria were set for inclusion: children aged 6.0 to 16.11 years, meeting the clinical diagnosis of TS (ICD-10: F95.2) with or without OCD, ADHD, anxiety, unipolar depressive disorders, rage attacks, or disruptive behavioral problems. 

#### 2.1.2. Exclusion Criteria

The exclusion criteria were defined as follows: other psychiatric conditions or developmental disorders not typically associated with TS (e.g., autism spectrum disorder, adjustment disorder, bipolar disorder, schizophrenia spectrum disorders); intelligence quotients (IQ) (Full-Scale) <70 according to the Wechsler scale of intelligence 4th edition (WISC-IV) [55]; brain malformations; and genetic syndromes. 

### 2.2. Testing Procedure

A week after intake, children who obtained a TS diagnosis, along with their parents, were asked to attend three testing sessions, 1.5 h in duration each, to perform cognitive testing and evaluate possible associated conditions and psychopathological symptoms. The evaluation was conducted simultaneously and separately with the parents and the child by two clinicians with solid experience in the administration of the selected study measures (detailed description of all study measures provided in the following paragraph). A cognitive evaluation was performed using the WISC-IV battery. A K-SADS-PL clinical interview [56], administered to both the parents and the child, was implemented for the identification of other psychiatric or neurodevelopmental conditions according to the DSM-5 criteria. The children were administered the YGTSS, the Children’s Yale–Brown Obsessive-Compulsive Scale (CY-BOCS) [57], the Children’s Depression Inventory (CDI) [58], and the Multidimensional Anxiety Scale for Children (MASC) [59]. Parents also completed the Conners Parent Rating Scale—revised (CPRS-R) [60] to rate ADHD symptom severity and were administered the YGTSS and CY-BOCS to gain information on parental perceptions of tics and OC symptom severity. As measure of quality of life and school functioning, the PEDsQol 4.0 [61], was implemented as both a self- and parent-report.

### 2.3. Measures

#### 2.3.1. WISC-IV

The WISC-IV consists of 10 core subtests providing four indexes of cognitive abilities standardized for sex and age. Global intelligence, as captured by the Full-Scale IQ (FSIQ) index, remains one of the single best predictors of academic and occupational success [62,63] and appears invariant and unbiased across gender, disability, and ethnic groups [64,65]. The FSIQ derives from the combination of the four indexes of the WISC-IV, reflecting the individual’s overall cognitive ability. The mean FSIQ standardized score is 100, with a standard deviation of ±15 points. Scores within one standard deviation around the mean are considered to reflect normal cognitive performance. The Verbal Comprehension Index (VCI) is the one of the scale’s sub-indexes which measures language expression and comprehension, as well as the ability of verbal reasoning to solve problems. The Perceptual Reasoning Index (PRI) reflects the ability to understand visual information and perform logical reasoning. The Working Memory Index (WMI) evaluates short-term memory and attention and, finally, the Processing Speed Index (PSI) indicates cognitive speed, but also relates to other cognitive factors, such as attention, as well as fine motor abilities. 

#### 2.3.2. YGTSS

The YGTSS is a reliable clinician-rated interview [66] that allows for the notation of tics currently experienced by the patient, based both on clinical observation and child and parent reports. Clinicians are asked to separately evaluate motor tics and phonic tics in terms of number, frequency, intensity, complexity, and interference on a 0–5 scale (with 5 indicating maximum severity). The total score is obtained by summing the scores from each dimension for both motor and phonic tics on a total scale (0–50 max.). Data from a large validation cohort of children with TS [67] have revealed the good to very good discriminant validity of the YGTSS, as well as acceptable internal consistency (Ω = 0.58 for YGTSS total tic score).

#### 2.3.3. CY-BOCS 

The children’s version of the Y-BOCS (CY-BOCS) is the most widely used measure to assess obsessive compulsive symptom severity. It is a semi-structured interview made up of 10 items rated on a 5-point Likert scale with a total score of 0–40 points. It evaluates the severity of obsessions and compulsions across five dimensions, frequency, interference, distress, resistance, and control, during the last week, and a score above 16 is generally considered indicative of the presence of OCD (16–23 = moderate severity; 24–40 = severe) [68].

#### 2.3.4. CPRS-R

The Conners Parent Rating Scale—Revised, short version (CPRS-R), is a proxy report used to assess ADHD symptom severity as well as behaviors that might be indicative of disruptive behavior disorders in children and adolescents ages 3 to 17 years. The Italian version was implemented for this study [69], consisting of 27 items on a 4-point Likert scale, summed to yield 4 subscales: Oppositional Problems, Cognitive Problems/Inattention, Hyperactivity, and ADHD index. Raw scores are transformed into T scores, and scores > 70 in the Cognitive, Hyperactivity, and ADHD index subscales offer maximal discrimination between child and adolescent psychiatric outpatients who were and were not diagnosed with ADHD [70,71].

#### 2.3.5. CDI

Depressive symptoms were evaluated through the Italian version of the Children’s Depression Inventory (CDI) [72], a 27-item self-report scale for children aged 8–17 years. The 27 items are scored on a 0–2-point Likert scale and investigate depressive symptoms across five areas (negative mood, interpersonal difficulties, negative self-esteem, ineffectiveness, and anhedonia). Scores above 20 are considered indicative of clinical depression, while those higher than 13 are considered an “alarm threshold” [72], proving efficient discrimination of children presenting with depressive spectrum disorders from youngsters without depressive disorders [73].

#### 2.3.6. MASC

The Italian version of the Multidimensional Anxiety Scale for Children (MASC) is a 39-item self-report scale for children ages 8 to 19 that covers 4 domains of anxiety symptoms [74]: Physical (tense/restless and somatic/autonomic problems), Social (humiliation/rejection, and public performance fears), Harm Avoidance (perfectionism and anxious coping), and Separation Anxiety. Single items are scored on a 0–3-point Likert scale, summed to their corresponding domain, and converted into T scores. Scores > 65 indicate significantly elevated levels of anxiety.

#### 2.3.7. K SADS-PL

The K-SADS-PL is a comprehensive interview allowing clinicians to diagnose current and past episodes of psychopathology and the presence of neurodevelopmental disorders in children and adolescents according to the DSM-5 criteria [56].

#### 2.3.8. PEDsQoL 

The PeDsQoL 4.0 is a modular self-report and parent-report scale assessing health-related quality of life in children and adolescents ages 2 to 18. It comprises 23 items covering four domains of the child’s functioning: Physical, Emotional, Social, and School. A 0–4-point response scale is used across the age group of 8 to 18 years (with 4 indicating “almost always a problem”), and items are reverse-scored and linearly transformed to a 0 to 100 scale (0 = 100, 1 = 75, 2 = 50, 3 = 25, 4 = 0), so that higher scores indicate better functioning. Acceptable reliability and validity for both patient self-reports and parent proxy reports was detected in the Italian validation study of the scale [75]. The self- and proxy-report measures for children aged over 8 years were implemented for this study.

### 2.4. Statistical Analysis

A descriptive analysis was conducted to characterize the sample. Quantitative variables were summarized by means and standard deviations (SDs), and categorical data by absolute frequencies and percentages. Pearson’s correlation analyses were carried out to verify possible co-variations between quantitative variables. Age; age at tic onset; YGTSS, CY-BOCS, CDI, MASC, and CPRS subscales; and WISC scores were all considered for a pairwise correlational analysis. A linear regression model was carried out using the FSIQ and WISC sub-indexes as dependent variables to evaluate the independent effect on cognitive performance of single comorbidities and disease duration. The model included, as independent variables, TS duration (i.e., the difference between age at evaluation and age at tic onset), age at tic onset, sex, familial history of tic disorders (Yes vs. No), and YGTSS, CY-BOCS, CPRS, CDI, and MASC scores. The Variance Inflation Factor (VIF) was computed to assess the presence of multicollinearity between the independent variables included in the model, which was suggested by VIF values greater than 5. Since a VIF value greater than 5 was observed for the CPRS ADHD index subscale, this variable was excluded from the model. R-squared values were computed to measure the percentage of variance of the dependent variable explained by the model. R-squared values around 0.13 could be considered as denoting a moderate effect size, while values about 0.26 or higher could be interpreted as large effect size [76]. The regression analysis results are presented as unstandardized regression coefficients (b), with significance levels and 95% confidence intervals (CIs), and standardized (beta) regression coefficients. Finally, to verify if cognitive functioning mediates the effect of the severity of comorbidities and tics on children’s QoL, mediation analyses were performed by structural equation modeling (maximum likelihood estimation method) using the Baron and Kenny approach to test mediation [77], including the FSIQ as a mediator; TS duration and YGTSS, CY-BOCS, CPRS, CDI, and MASC scores as independent variables; and the PEDsQoL Total, Physical, Emotional, Social, and School subscales as dependent variables in separate analyses. Coefficients and significance levels were calculated for the direct, indirect, and total effects of all variables included in the models. Indirect (i.e., mediating) effects were evaluated with 95% confidence intervals according to both the Sobel and Monte Carlo methods. The results of both methods were consistent; thus, we report coefficients estimated according to Sobel. The data were all controlled for medication status, which was entered as a covariate in the model, and all statistical analyses were performed using the Stata Software, release 16 [78].

## 3. Results

### 3.1. Clinical and Demographic Characteristics

A total of 80 participants diagnosed with TS were included in this study. The demographic data are summarized in Table 1. The mean age was 10.7 years (±2.3 SD), and 90% of participants were males.

A familial history of tic disorders was reported in 52.5% of cases. Regarding pharmacological treatment, 14 (17.5%) patients were already on medication—prescribed elsewhere—at the time of evaluation: nine (11.3%) were receiving Aripiprazole for their tics, two (2.5%) patients were on active SSRI treatment for obsessive compulsive or anxious symptomology, and three (3.8%) were receiving combination therapy (Aripiprazole plus SSRI). Only two children had previously received behavioral intervention for tics. As regards comorbidities, 63.8% of children presented at least one comorbidity at the time of psychiatric evaluation. In detail, 23.8% were diagnosed with comorbid ADHD, 28.8% with OCD, 12.5% with depression, and 26.3% with anxiety disorders. Full details on the comorbidity profiles are listed in Table 1. The children’s psychometric data are summarized in Table 2.

### 3.2. Cognitive Performance 

The mean Full-Scale Intelligence Quotient (FSIQ) score among our patients was 108 (SD = ±15). The FSIQ scores were between 71 and 85 in five patients (6.3%), indicating borderline mental functioning. A detailed description of the WISC-IV indexes is displayed in Table 3.

### 3.3. Correlations

#### 3.3.1. Parental vs. Child YGTSS, CY-BOCS, and PEDsQoL Score Correlation

A close relation was detected between the parents’ and children’s YGTSS and CY-BOCS (Table 4) scores, suggesting a similar perception of tic and obsessive-compulsive symptom severity by child and family. Similarly, the children’s perception of their QoL was comparable to that of parents filling in the proxy-report version of the questionnaire (Table 4). Therefore, unless specified, the YGTSS, CY-BOCS, and PEDsQoL scores refer hereinafter to those obtained from the patients only. 

#### 3.3.2. Correlation between Study Measures

No significant correlations were found between the YGTSS and CY-BOCS scores, whereas such measures, when considered separately, appeared positively correlated to the MASC and CDI scores (Table 5).

#### 3.3.3. Correlation between Study Measures and Cognitive Indexes 

Several significant negative correlations were detected between both age and the CPRS subscales with the WISC indexes (Table 6). Age showed negative correlation with the total IQ (r = −0.29, *p* = 0.009) and WMI (r = −0.36, *p* = <0.001). The total IQ was negatively influenced by all CPRS subscales. Among these, Cognitive Problems showed the greatest significant negative correlations with all WISC indexes, with medium correlational strength. 

Only the parents’ but not patients’ CY-BOCS scores were negatively correlated with the WMI scores (r = −0.32, *p* = 0.005).

A correlational analysis failed to reveal other significant variations in the WISC according to the age at tic onset and the patient’s YGTSS, CY-BOCS, CDI, and MASC scores.

### 3.4. Multiple Linear Regression Analysis

To verify if comorbidities, tic severity, and disease duration were associated with significant variations in cognitive performance in children with TS, multiple regression analyses were conducted. The results (Table 7) showed that a greater disease duration was predictive of a lower FSIQ (b = −2.07, *p* = 0.013), VCI (b = −1.97, *p* = 0.026), and WMI (b = −1.81, *p* = 0.037), while greater attentive problems, as measured by the CPRS Cognitive Problems subscale, were associated with a worse PSI (b = −0.42, *p* = 0.014). Moreover, greater depressive symptoms predicted a lower FSIQ (b = −0.86, *p* = 0.008) and PRI (b = −0.89, *p* = 0.02). Conversely, higher levels of anxiety symptoms as captured by the MASC were predictive of better cognitive performance in terms of the FSIQ (b = 0.61, *p* = 0.001) and all WISC subscales. The model accounted for 45% of the variance.

### 3.5. Total IQ as Mediator of the Relationship between Clinical Severity of TS and QoL

Finally, based on previous results, we verified whether the overall cognitive profile captured by the FSIQ mediated the effect on well-being perceived by the children according to the scores given by the PEDsQoL. 

**Figure 1:**
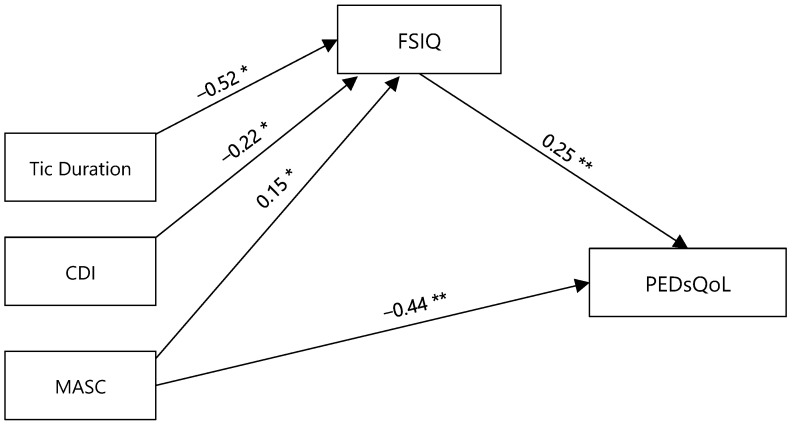
Regression coefficients for the relationship between PEDsQoL and independent variables of TS clinical severity as mediated by the Full-Scale Intelligence Quotient (FSIQ). * *p* < 0.05, ** *p* < 0.01.

As can be seen in Figure 1, the results of the mediation analysis showed that the total effects of the CDI scores and MASC scores on the total PEDsQoL score were significant. However, for the CDI scores, the direct effect was non-significant, thus revealing that the effect of the CDI scores on the overall QoL was mediated completely by the FSIQ. As for MASC scores, these were both directly linked to a worse QoL, as well as being indirectly associated, by means of their effect on the FSIQ, to better QoL ratings. The disease duration was influential on QoL, but only through an indirect pathway, i.e., by having a negative effect on the FSIQ. 

Regarding the different PEDsQoL subscales, only the Social Functioning and School Functioning QoL scores were mediated by the FSIQ, whereas the Physical Functioning and Emotional Functioning QoL subscales were not. Specifically, the CDI scores were negatively influential on Social functioning through an indirect effect on the FSIQ, whereas the MASC scores had a positive effect on Social and School Functioning through the effect played on FSIQ.

The MASC and CDI scores also had a direct effect on Emotional Functioning QoL that was independent from the FSIQ (indirect effect, non-significant).

## 4. Discussion

The present study offers novel insights into how different clinical features of TS impact the cognitive functioning and QoL of children and adolescents. Our results emphasize that in pediatric TS, depression, anxiety, and disease duration largely contribute to both the QoL and the cognitive functioning of children, while ADHD results in poorer cognitive performance in processing speed abilities. 

Although largely associated, in the general population, with variable cognitive deficits, particularly in executive functioning [79,80], depression in children and adolescents with TS often remains under-recognized in clinical practice, with clinical studies often failing to report depressive symptomatology. However, children and adolescents with TS experience affective symptoms significantly more often than their healthy peers [14], and up to 76% of patients attending specialist clinics show mild to severe depressive symptoms [81]. The relationship between TS and depression appears to be multifactorial rather than simply reactive to the frustration and distress caused by chronic tics [81]. Our study expands the knowledge on this issue by showing that in youngsters with TS, depression severity is predictive of a reduction in the total IQ and processing speed index, which in turn negatively influences the QoL perceived by children. Our findings parallel those by Hovik et al. [82] that pointed out that depression is linked to worse cognitive performance in TS, regardless of other comorbidities. Moreover, we highlight that depression acts on reducing the well-being of children not only by influencing their emotional functioning, but also by impairing their cognition, a key QoL contributor. Taking together our results and the fact that depressive symptoms in youths with TS appear to increase over time [83], we strongly advocate for the routine assessment and treatment of depression in children and adolescents.

The influence of anxiety on cognition and QoL in our sample was more nuanced. On the one hand, an anxious symptomatology predicted better IQ values and performance in all cognitive domains except processing speed. On the other hand, the overall effect of anxiety on QoL was negative, and this was driven by the role played by anxiety in the emotional and social functioning of children, as revealed by our mediation analysis. The relationship between anxiety and cognition is complex. While a large body of research has shown its negative impact on cognitive activities [84,85,86], anxiety may not always interfere with performing a demanding task, or may even be reduced by it [87,88,89]. Of note, although a categorial diagnosis of any type of anxiety disorder was present in 26.3% of cases, only 5% of our cohort reported anxiety symptoms that reached the clinical threshold of significance according to the MASC. Therefore, the observed influence of anxiety on cognitive performance may largely be due to subclinical anxiety levels, which seem to have a positive influence on cognitive abilities. This is also in line with prior studies showing that elevated physiological anxiety, when accompanied by low levels of worry, may predict better IQ performance in children as well as in the elderly [90,91]. Overall, these findings cautiously suggest that in certain tasks with high attentional demands (such as those required by the WISC-IV), preparatory mechanisms associated with non-clinical levels of arousal, like increased vigilance, may facilitate error monitoring and rapid response and promote better performance [86,88]. However, our results show that, overall, anxiety exerts a detrimental impact on the daily functioning of children with TS by reducing their self-esteem and emotional well-being (b = −0.96, *p* = 0.007) and their perceived competence in social interactions (b = −0.66, *p* = 0.001).

Unsurprisingly, our study replicates a wealth of TS studies pointing to worse cognitive performance and lower IQ in children with concomitant ADHD and can be interpreted similarly [43,44,45,46,47,48]. Of all cognitive domains, inattentive symptom severity was the only clinical feature of ADHD in our cohort that significantly predicted a reduction in processing speed. Processing speed represents the pace at which information is received, elaborated, and replied to by the brain. This ability is important for presumably all functions, including behavioral regulation. Indeed, in children with ADHD, reduced processing speed has been repeatedly proposed as a useful indicator of worse behavioral functioning [92,93] and a risk factor for later peer problems during adolescence [94]. Moreover, processing speed deficits have been posited as a potential neuropsychological marker of ADHD that may distinguish inattention due to ADHD from that due to other mental health conditions, such as depression [95,96]. Altogether, our results suggest that assessing the cognition in children with TS—with a particular focus on processing speed—could be even more important in the presence of concomitant ADHD, given these implications for the cognitive and behavioral functioning of children. 

No significant role for obsessive compulsive symptoms was detected in our study, neither for cognition nor for QoL. Our results contrast a larger base of studies that point toward better cognitive abilities in individuals with TS and comorbid OCD as compared to the WISC norm [46,97]. However, multilayered evidence exists regarding cognitive functioning in OCD in general. On the one hand, many studies have suggested abnormalities in cognitive flexibility and memory in both patients and their unaffected relatives (e.g., [98,99,100]); on the other hand, data from the largest metanalysis available to date revealed no substantial cognitive impairments in children with OCD [101]. Such heterogeneity may have arisen from the frequent consideration of OCD as a homogeneous condition by the extant neuropsychological literature. Prior studies have mainly examined state severity measures of obsessive-compulsive symptoms (e.g., the CY-BOCS) as possible moderators of patients’ cognitive performance and may have lacked consideration of the role of other clinical factors of the disorder, such as disease duration, the type of obsessions/compulsions, and comorbid conditions [102,103,104]. Further, regarding the association of TS and OCD specifically, emerging evidence points to different neurobiological underpinnings in OCD with TS and without it [105,106,107]. Hypothetically, different neural correlates of OCD in the context of TS as compared to “pure” OCD might further be associated with differences in neuropsychological profiles. This possibility awaits future studies with well-defined comorbidity profiles to increase insight into the role of OCD on cognition in children with and without chronic tics.

Currently, it is unknown whether and how the persistence of tics, i.e., disease duration, contributes to long-term outcomes in TS. In our study, the overall duration of tics was independently predictive of a lower IQ, verbal reasoning, and working memory. Thus, despite typical tic fluctuations over time and the presence of other comorbidities, children experiencing tics over longer periods of time showed poorer cognitive abilities. Furthermore, cognitive abilities moderated the relationship between disease duration and QoL, such that part of the effect of disease duration on well-being was mediated by the effect of disease duration on IQ. Regarding the age at tic onset, although prior studies with different methodologies have pointed out that an earlier TS onset may be associated with a lower IQ [44,49], our study revealed no such effect. Therefore, the observed effect of disease duration on cognition in our study cannot be solely attributed to an earlier age at tic onset, but rather to the cumulative effect of having tics for greater amounts of time and, putatively, to the persistence of disease-related biological changes and the development of higher clinical complexity that a longer disease duration may involve. Greater consideration in future studies of disease duration is crucially warranted to shed light on the effect of tic persistence on cognition and functional outcomes in people with lived experience of TS.

This study should be interpreted in light of some limitations. First, we cannot exclude that our results might be partially due to a selection bias. Specifically, the small number of participants who reported clinical or moderate/severe symptoms of anxiety and obsessions/compulsions undoubtedly diminished the power of our analyses (i.e., fewer data available on WISC scores from children with more severe symptoms). Secondly, the reduced representation of female participants in our cohort further limits the generalizability of our results to the overall TS population. Moreover, although we controlled our results for the patient’s medication status (present/absent), the medication type (e.g., antipsychotics, SSRI, both) and treatment duration were different across participants. Therefore, no conclusive considerations can be drawn regarding their influence on cognitive profiles. Next, our findings are based on cross-sectional analyses and, therefore, do not explain if symptom fluctuations over time may determine different effects on the cognitive functioning of patients in the long term. Lastly, during the clinical assessment, no systematic information was collected on the patients’ socioeconomic statuses. Thus, it was not possible to control the IQ scores for such an important factor [108,109]. 

Despite such limitations, our study is of particular use for understanding the impact of the TS clinical spectrum on life quality. Depression deeply affects the cognitive functioning of children living with TS. Depression also impacts on their perceived QoL through the indirect effect played by depressive symptomatology on cognition. Likewise, a greater disease duration and ADHD inattentive symptoms are both detrimental on cognitive performance, but only disease duration was influential on children’s QoL ratings. Conversely, mild forms of anxiety (or trait anxiety) may slightly enhance cognitive performance in TS but not QoL, while OCD was not clearly influential on cognitive outcomes. Altogether, we highlight that disease duration is critical for explaining the variance in cognitive performance in TS, and that a greater duration of tics translates into a poorer perceived well-being of children. Comorbidities, particularly depression, need to be considered as sensitive risk factors for educational and academic underachievement [110] and the need for additional support [111] in this specific population.

Based on our results, it is central for clinicians to promptly identify and quantify comorbidities in children and adolescents with TS to start interventions aimed at their treatment in a timely manner. This may promote a risk reduction strategy against their detrimental consequences on cognitive functioning and overall well-being. Moreover, the finding regarding the impact of disease duration on cognition deserves further consideration in both the clinical and research fields since it raises new practical and ethical questions about treating children and adolescents with chronic tics. There are complex issues surrounding medication use in this age group, including the consideration of when to start medication and the potential impact of sustained medication use on the developing brain. Still, very little is known about TS’s developmental trajectories and their determinants. Future studies explaining why some individuals develop milder forms of TS while others do not are critically needed to decide the timing of treatment start and end and improve the well-being of all patients.

## Figures and Tables

**Table 1:** Demographic and clinical characteristics of the study population.

Sex	*N* (%)
Males	72 (90.0)
Females	8 (10.0)
**Age**	
6–7 years	7 (8.8)
8–9 years	27 (33.8)
10–11 years	25 (31.3)
12–13 years	12 (15.0)
14–16 years	9 (11.3)
**Familial history of tics**	
	42 (52.5)
**Pharmacological Treatment**	
Any treatment	14 (17.5)
SSRI	2 (2.5)
Aripiprazole	9 (11.3)
SSRI + Aripiprazole	3 (3.8)
**Comorbidities**	
ADHD	19 (23.8)
OCD	23 (28.8)
Depression	10 (12.5)
Anxiety	21 (26.3)
ADHD + OCD	3 (3.8)
ADHD + Depression	2 (2.5)
ADHD + Anxiety	6 (7.5)
ADHD + OCD + Depression	/
ADHD + OCD + Anxiety	2 (2.5)
OCD + Depression	4 (5.0)
OCD + Anxiety	10 (12.5)
Depression + Anxiety	8 (10.0)

Data presented as absolute values (percentage). SSRI = selective serotonin reuptake inhibitor.

**Table 2:** Psychometric characteristics of the sample.

YGTSS			
*Scores*	*Mean*	*SD*	
Total score	16.8	8.2	
Motor tics	11.2	4.7	
Vocal tics	5.7	5.3	
*Score severity*	*N*		
mild (<14)	29		
moderate (15–34)	50		
severe (≥35)	1		
**CY-BOCS**			
*Scores*	*Mean*	*SD*	
Total score	7.9	7.3	
Compulsions	3.1	3.9	
Obsessions	4.8	4.3	
*Score severity*	*N*		
subclinical (<8)	43		
mild (8–15)	23		
moderate (16–23)	11		
severe (24–31)	1		
extremely severe (32–40)	0		
**MASC**			
*Score*	*Mean*	*SD*	
Total score	10.2	6.0	
*Score severity*	*N*		
non-clinical (<60)	56		
borderline (60–69)	11		
clinical (≥70)	4		
**CDI**			
*Score*	*Mean*	*SD*	*N in the clinical range*
Total score	10.2	6.0	16
**CPRS**			
*Scores*	*Mean*	*SD*	*N in the clinical range*
Oppositionality	56.6	16.1	17
Cognition	58.0	16.1	16
Hyperactivity	56.8	15.0	17
ADHD index	60.8	15.1	21

*N* = number of patients in absolute values.

**Table 3:** WISC-IV mean scores, standard deviation (SD), and score range.

WISC-IV Index	Mean	SD	Score Range
FSIQ	108	15	78–139
VCI	112	14	72–138
PRI	109	17	67–143
WMI	105	15	67–130
PSI	95	15	53–138

FSIQ: Full-Scale Intelligence Quotient, VCI: Verbal Comprehension Index, PRI: Perceptual Reasoning Index, WMI: Working Memory Index, PSI: Processing Speed Index.

**Table 4:** Correlations between children’s and parent’s YGTSS and CY-BOCS.

		r	*p*
YGTSS	Total score	0.49	<0.001
	motor tics	0.59	<0.001
	vocal tics	0.42	<0.001
	impairment score	0.51	<0.001
CYBOCS	Total score	0.48	<0.001
	obsessions	0.47	<0.001
	compulsions	0.43	<0.001
PEDsQoL	Total score	0.52	<0.001
	Physical functioning	0.53	<0.001
	Emotional functioning	0.46	<0.001
	Social functioning	0.49	<0.001
	School functioning	0.50	<0.001

**Table 5:** Correlations between tic and obsessive compulsive symptom severity with CDI and MASC.

		YGTSS				CY-BOCS		
		Total	Motor Tics	Vocal Tics	Impairment Score	Total	Obsessions	Compulsions
*CDI*	r	0.41	0.31	0.35	0.31	0.09	0.07	0.09
	*p*	<0.001 *	0.008 *	0.002 *	0.008 *	0.431	0.543	0.421
*MASC total*	r	0.38	0.28	0.34	0.26	0.31	0.25	0.31
	*p*	0.001 *	0.017 *	0.003 *	0.026 *	0.01 *	0.033 *	0.014 *

* Significant *p*-value.

**Table 6:** Correlation between WISC indexes, CPRS subscales, and age.

		WISC-IV				
		FSIQ	VCI	PRI	WMI	PSI
**CPRS**						
Oppositionality	r	−0.29	−0.23	−0.18	−0.26	−0.28
	*p*	0.009 *	0.041 *	0.011	0.024 *	0.013 *
Cognitive Problems	r	−0.43	−0.29	−0.24	−0.39	−0.47
	*p*	<0.001 *	0.008 *	0.031 *	<0.001 *	<0.001 *
Hyperactivity	r	−0.34	−0.26	−0.22	−0.33	−0.32
	*p*	0.002 *	0.021 *	0.051	0.002 *	0.004 *
ADHD index	r	−0.37	−0.28	−0.22	−0.33	−0.45
	*p*	<0.001 *	0.012 *	0.054	0.003 *	<0.001 *
**AGE**	r	−0.29	−0.20	−0.17	−0.36	−0.17
	*p*	0.009 *	0.072	0.0142	<0.001 *	0.139

* Significant *p*-value. FSIQ: Full-Scale Intelligence Quotient, VCI: Verbal Comprehension Index, PRI: Perceptual Reasoning Index, WMI: Working Memory Index, PSI: Processing Speed Index.

**Table 7:** Influence on the different cognitive indexes of the clinical features of TS.

	FSIQ			VRI			PRI			WMI			PSI		
	b (Beta)	*p*	95% CI	b (Beta)	*p*	95% CI	b (Beta)	*p*	95% CI	b (Beta)	*p*	95% CI	b (Beta)	*p*	95% CI
*TS duration years*	**−2.069** **(−0.299)**	0.013 *	**−3.668** **to** **−0.449**	**−1.973** **(−0.308)**	0.026 *	**−3.696** **to** **−0.249**	−1.649 (−0.216)	0.095	−3.595 to 0.296	**−1.809** **(−0.269)**	0.037 *	**−3.507** **to** **−0.112**	−0.293 (−0.041)	0.742	−2.073 to 1.485
*Age at TS onset*	−0.064 (−0.010)	0.931	1.531 to 1.402	−0.452 (−0.074)	0.578	−2.066 to 1.163	0.712 (0.100)	0.421	−1.045 to 2.468	−0.343 (−0.055)	0.657	−1.883 to 1.196	−0.523 (−0.079)	0.519	−2.138 to 1.091
*Sex*	7.154 (0.143)	0.231	4.682 to 18.991	−2.102 (−0.044)	0.748	−15.131 to 10.927	8.715 (0.159)	0.223	−5.461 to 22.890	3.932 (0.081)	0.529	−8.493 to 16.356	11.476 (0.224)	0.083	−1.555 to 24.507
*Familial history*	−1.058 (−0.035)	0.746	7.570 to 5.454	−1.763 (−0.062)	0.624	−8.931 to 5.405	−1.897 (−0.057)	0.628	−9.695 to 5.901	2.078 (0.071)	0.545	−4.757 to 8.913	0.365 (0.012)	0.919	−6.804 to 7.534
*YGTSS total*	0.072 (0.039)	0.741	0.359 to 0.503	−0.096 (−0.056)	0.686	−0.571 to 0.378	0.308 (0.155)	0.237	−0.208 to 0.824	−0.077 (−0.044)	0.734	−0.530 to 0.375	0.294 (0.158)	0.220	−0.181 to 0.769
*CYBOCS total*	−0.163 (−0.082)	0.481	0.622 to 0.297	0.172 (0.092)	0.498	−0.334 to 0.678	−0.291 (−0.133)	0.294	−0.841 to 0.259	−0.303 (−0.157)	0.214	−0.785 to 0.180	−0.217 (−0.107)	0.393	−0.723 to 0.289
*CPRS Oppositionality*	−0.048 (−0.048)	0.776	0.387 to 0.290	−0.029 (−0.031)	0.876	−0.402 to 0.343	−0.044 (−0.040)	0.830	−0.449 to 0.361	0.163 (0.168)	0.363	−0.193 to 0.518	−0.031 (−0.030)	0.869	−0.403 to 0.342
*CPRS Cognitive Problems*	−0.170 (−0.161)	0.237	0.455 to 0.115	−0.069 (−0.069)	0.662	−0.382 to 0.245	−0.006 (−0.006)	0.970	−0.347 to 0.335	−0.120 (−0.118)	0.426	−0.419 to 0.179	**−0.419** **(−0.374)**	0.014 *	**−0.751** **to** **−0.086**
*CPRS Hyperactivity*	−0.216 (−0.215)	0.205	0.554 to 0.121	−0.206 (−0.217)	0.272	−0.578 to 0.166	−0.261 (−0.237)	0.201	−0.666 to 0.143	−0.346 (−0.357)	0.055	−0.701 to 0.008	−0.060 (−0.058)	0.748	−0.432 to 0.312
*CDI total*	**−0.860** **(−0.349)**	0.008 *	**−1.484** **to** **−0.237**	−0.634 (−0.278)	0.061	−1.297 to 0.029	**−0.893** **(−0.328)**	0.020 *	**−1.642** **to** **−0.143**	−0.581 (−0.242)	0.080	−1.234 to 0.072	−0.459 (−0.182)	0.181	−1.137 to 0–219
*MASC total*	**0.605** **(0.463)**	0.001 *	**0.252** **to** **0.957**	**0.427** **(0.353)**	0.026 *	**0.053** **to** **0.802**	**0.611** **(0.423)**	0.005 *	**0.188** **to** **1.034**	**0.455** **(0.358)**	0.017 *	**0.085** **to** **0.823**	0.294 (0.218)	0.130	−0.090 to 0.677

b is unstandardized and Beta is standardized regression coefficient. * Significant *p*-values (also highlighted in bold). CI = confidence interval of regression coefficient b. FSIQ = Full-Scale Intelligence Quotient; VRI = Verbal Reasoning Index; PRI = Perceptual Reasoning Index; WMI = Working Memory Index; PSI = Processing Speed Index.

## Data Availability

The raw data supporting the conclusions of this article will be made available upon reasonable request to the authors, without undue reservation. The data are not publicly available due to privacy reasons.
